# Supplementary material for: Carbon and Nitrogen Stable Isotopic Profiling of Chimpanzees and Monkeys in Kalinzu Forest Reserve, Uganda
Source: Am J Primatol. 2026 Jan 20;88(1):e70114. doi: 10.1002/ajp.70114 (PMC12820444; doi:10.1002/ajp.70114)
Supplement: Supplementary file 1 — kalinzuhair2_si. [file AJP-88-e70114-s001.pdf]

Supporting Information

Carbon and nitrogen stable isotopic profiling of chimpanzees and monkeys in Kalinzu Forest Reserve, Uganda

Takumi Tsutaya, Natsumi Aruga, Naoto F. Ishikawa, Yoko Sasaki, Haruka Kitayama, Minoru Yoneda, Nana O. Ogawa, Naohiko Ohkouchi, Chie Hashimoto

Table of Contents

Supporting Text S1 2

Supporting Figures 3

Supporting Tables 5

Supporting References 21

Supporting Text S1

Relationship between rainfall and stable isotope ratios

Linear regression models were applied to predict the relationships between mean annual rainfall and average carbon and nitrogen stable isotope ratios of plants and hairs of chimpanzees in various sites. The predicted relationships are shown below:

- Plant $\delta^{13}C$ (‰) = –3.261e^{-3} × Rainfall (mm) – 22.81, R^2 = 0.6783
- Hair $\delta^{13}C$ (‰) = –1.683e^{-3} × Rainfall (mm) – 19.97, R^2 = 0.4065
- Plant $\delta^{15}N$ (‰) = 6.139e^{-4} × Rainfall (mm) + 2.65, R^2 = 0.108
- Hair $\delta^{15}N$ (‰) = 1.538e^{-3} × Rainfall (mm) + 3.96, R^2 = 0.0827

Supporting Figures

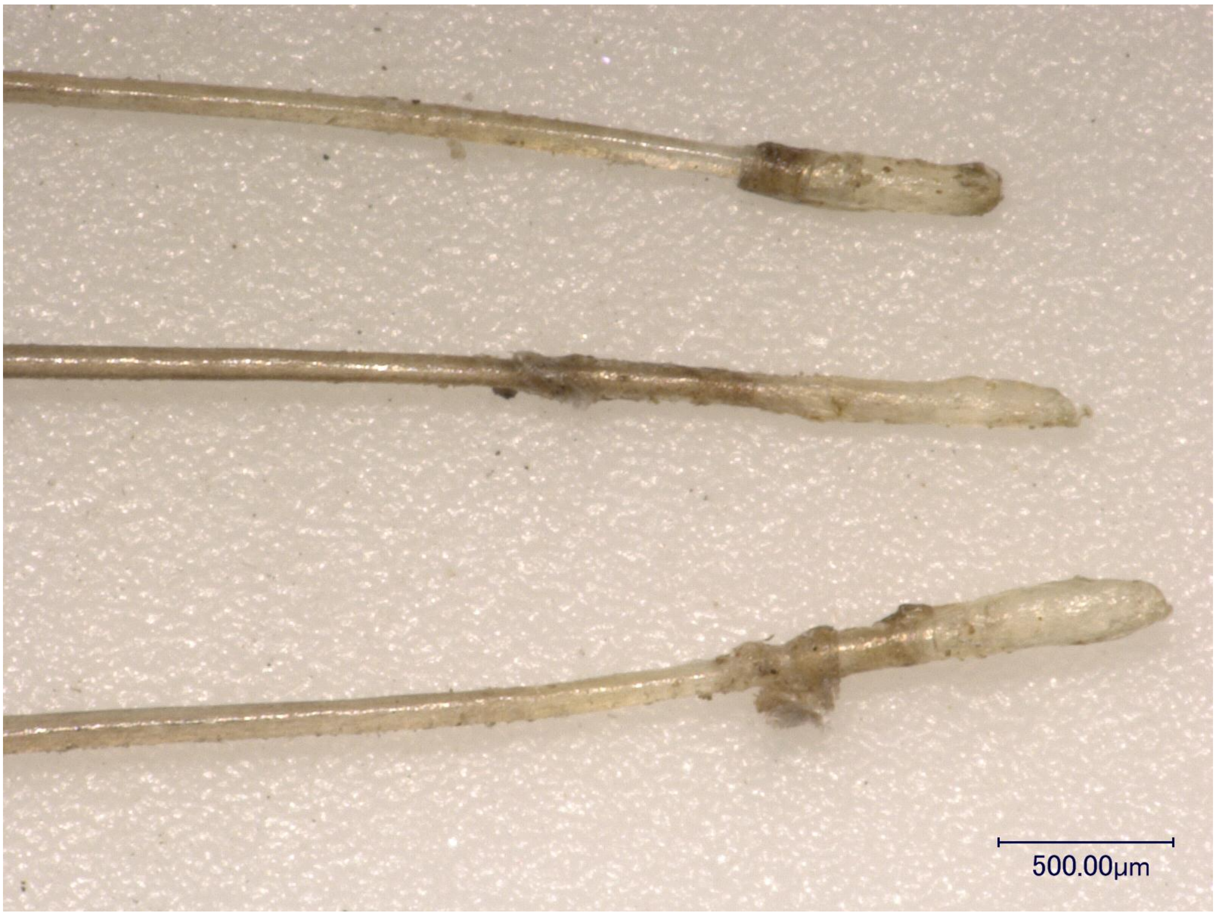

Supporting Figure S1. Microscopic photo of hair follicles of a male chimpanzee (Kobo).

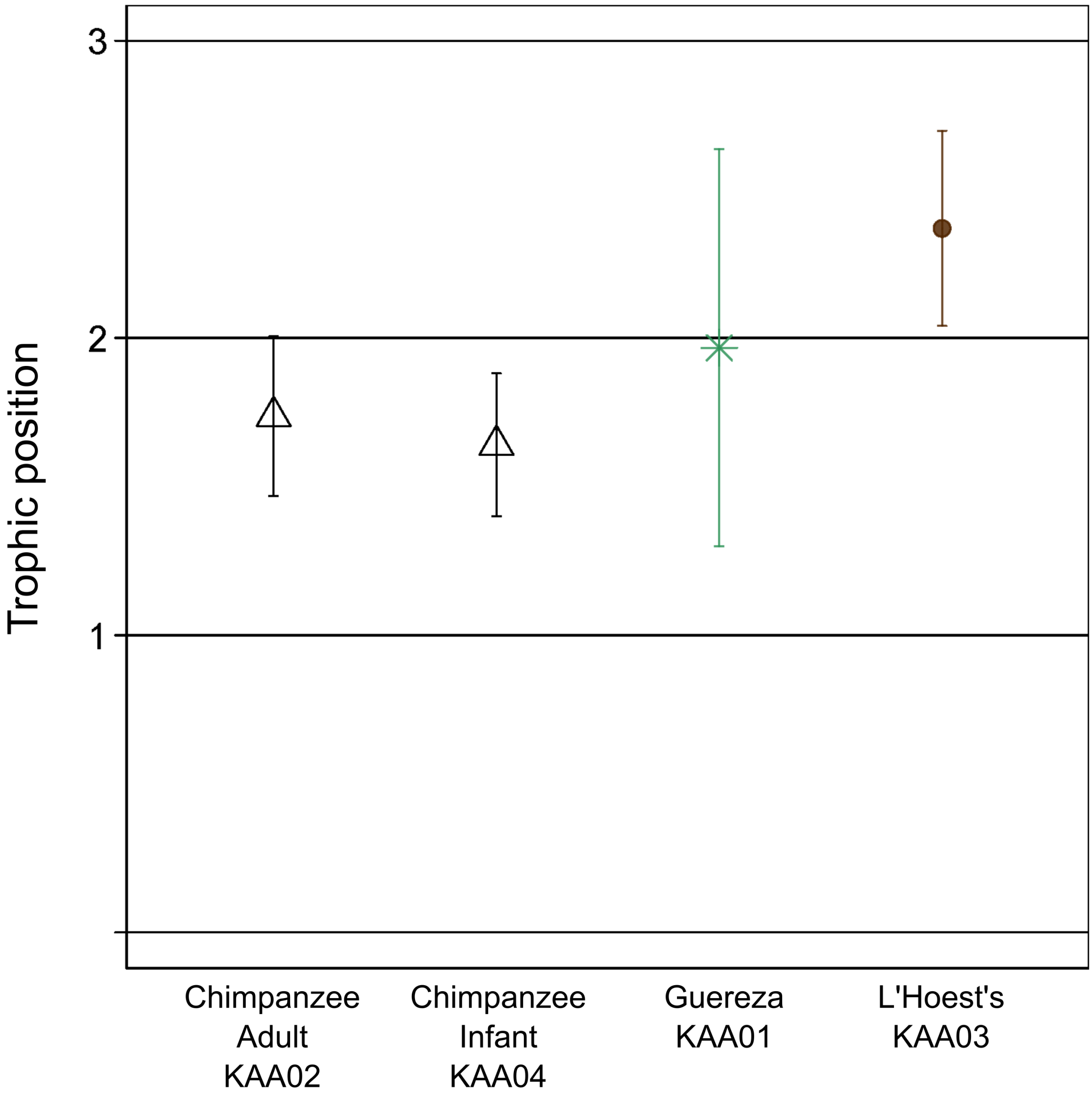

Supporting Figure S2. Calculated TP of primate species in Kalinzu. The error bars represent one standard deviation range of propagated uncertainties.

Supporting Tables

Supporting Table S1. Stable isotopic results of primate hairs from the Kalinzu Forest Reserve. The carbon and nitrogen weight proportions of the samples (%C and %N) and the atomic carbon to nitrogen ratios (C/N) were also shown.

ID	Date	Species	Individual	Age	Sex	$\delta^{13}C$	$\delta^{15}N$	%C	%N	C/N
KAA17	2015-01-06	Chimpanzee	Gai, Gaia, Gal, or Gawoe	Non-infant	F	-22.7	6.9	39.2	12.7	3.6
KAA22	2015-01-06	Chimpanzee	Shoko	Non-infant	F	-23.4	6.1	35.8	12.1	3.5
KAA23	2015-01-16	Chimpanzee	Minny	Non-infant	F	-23.4	5.5	39.1	13.4	3.4
KAA06	2013-07-23	Chimpanzee	Tange	Non-infant	M	-23.5	6.8	38.9	13.1	3.5
KAA02*	2013-07-30	Chimpanzee	Kobo	Non-infant	M	-24.4	7.0	45.3	15.4	3.4
KAA27	2015-02-02	Chimpanzee	Buru	Non-infant	M	-23.4	5.9	42.2	14.0	3.5
KAA30	2015-02-19	Chimpanzee	Goku, Prince, or Deo	Non-infant	M	-24.5	5.7	42.8	13.3	3.8
KAA04	2015-03-01	Chimpanzee	–	Infant	M	-24.1	6.3	40.7	14.3	3.3
KAA32	2015-05-13	Chimpanzee	Gure	Non-infant	M	-24.1	6.2	45.0	16.0	3.3
KAA40	2015-11-25	Chimpanzee	Goku	Non-infant	M	-24.7	5.9	41.8	13.2	3.7
KAA42	2015-12-03	Chimpanzee	Gure or Ponta	Non-infant	M	-25.4	6.8	44.3	13.7	3.8
KAA36	2015-04-16	Blue monkey	–	Infant	–	-24.5	7.7	46.2	15.6	3.5
KAA33	2015-06-11	Blue monkey	–	Non-infant	–	-24.5	7.1	47.0	16.8	3.3
KAA38	2015-07-23	Blue monkey	–	Non-infant	F	-24.6	6.8	47.9	16.9	3.3
KAA43	2015-12-07	Blue monkey	–	Infant	–	-22.9	7.9	49.3	16.1	3.6
KAA44	2017-02-23	Blue monkey	–	Non-infant	–	-23.3	7.8	41.5	13.8	3.5
KAA18	2015-01-06	Guereza	–	Non-infant	–	-21.6	9.0	39.8	13.6	3.4
KAA20	2015-01-15	Guereza	–	Non-infant	–	-21.2	9.3	38.5	12.9	3.5
KAA25	2015-01-21	Guereza	–	Non-infant	–	-21.1	7.5	39.9	13.4	3.5
KAA26	2015-01-30	Guereza	–	Non-infant	–	-21.3	8.5	39.8	13.9	3.3

KAA01	2015-03-08	Guereza	–	Non-infant	–	-22.7	7.7	42.8	14.5	3.4
KAA41	2015-12-01	Guereza	–	Infant	–	-20.8	11.1	48.9	17.3	3.3
KAA45	2017-05-25	Guereza	–	Non-infant	–	-22.9	9.6	45.3	15.6	3.4
KAA03	2015-03-01	L'Hoest's monkey	–	Non-infant	–	-21.8	11.3	45.5	16.0	3.3
KAA34	2015-07-13	Red-tailed monkey	–	Non-infant	–	-24.0	7.5	45.7	16.2	3.3
KAA39	2015-10-05	Red-tailed monkey	–	Non-infant	F	-25.2	7.9	48.9	16.7	3.4

Supporting Table S2. Stable isotopic results of food plants from the Kalinzu Forest Reserve. The carbon and nitrogen weight proportions of the samples (%C and %N) and the atomic carbon to nitrogen ratios (C/N) were also shown.

ID	Species	Part	Stratification	Date	$\delta^{13}C$	$\delta^{15}N$	%C	%N	C/N	Chimpanzee	Blue	Guereza	L'Hoest's	Red-tailed
K2-F062	*Acacia montigena*	Leaves	Gap	2015-02-27	-27.8	4.0	44.9	5.1	10.2	0	1	0	1	1
K2-F103	*Acacia montigena*	Leaves	Gap	2015-03-03	-30.6	6.5	47.2	4.9	11.3	0	1	0	1	1
K3-F045	*Acacia montigena*	Leaves	Gap	2015-11-27	-31.6	3.1	43.6	3.5	14.6	0	1	0	1	1
K3-F060	*Acacia montigena*	Leaves	Gap	2015-12-04	-29.8	5.9	45.7	5.4	9.8	0	1	0	1	1
K1-F013	*Aframomum mildbraedii*	Pith	Understory	2013-07-26	-35.4	1.1	42.1	1.1	45.2	1	0	0	1	0
K1-F014	*Aframomum mildbraedii*	Pith	Understory	2013-07-26	-32.7	5.0	44.5	2.6	20.1	1	0	0	1	0
K1-F030	*Aframomum mildbraedii*	Pith	Understory	2013-07-29	-32.0	3.7	45.4	1.3	41.3	1	0	0	1	0
K1-F045	*Agelaea pentagyna*	Leaves	Subcanopy	2013-07-29	-33.3	1.7	44.8	3.9	13.3	0	0	1	0	0
K1-F021	*Antiaris toxicaria*	Leaves	Crown	2013-07-26	-32.6	1.1	44.4	3.6	14.3	1	0	0	0	0
K1-F043	*Antiaris toxicaria*	Leaves	Crown	2013-07-29	-34.7	1.7	39.1	2.6	17.5	1	0	0	0	0
K2-F033	*Celtis durandii*	Fruits	Crown	2015-02-26	-26.1	4.3	49.4	3.2	18.3	1	1	1	1	1
K2-F064	*Celtis durandii*	Fruits	Crown	2015-02-27	-25.0	1.8	50.0	3.0	19.5	1	1	1	1	1
K3-F065	*Celtis durandii*	Fruits	Crown	2015-12-07	-25.0	3.1	40.5	2.4	19.8	1	1	1	1	1
K3-F066	*Celtis durandii*	Fruits	Crown	2015-12-07	-23.7	2.0	42.3	2.9	17.2	1	1	1	1	1
K1-F023	*Craterispermum laurinum*	Leaves	Subcanopy	2013-07-27	-32.9	4.7	36.3	2.7	15.9	0	1	0	1	1
K1-F042	*Craterispermum schweinfurthii*	Fruits	Subcanopy	2013-07-29	-28.0	2.5	45.5	1.6	32.9	1	1	0	0	1
K1-F002	*Dasylepis eggelingii*	Fruits	Subcanopy	2013-07-22	-33.2	4.3	45.3	1.3	40.0	0	0	0	1	0
K1-F035	*Dasylepis eggelingii*	Fruits	Subcanopy	2013-07-29	-30.4	3.3	45.3	2.7	20.0	0	0	0	1	0
K1-F036	*Dasylepis eggelingii*	Fruits	Subcanopy	2013-07-29	-34.0	3.1	42.9	2.0	25.6	0	0	0	1	0
K1-F001	*Drypetes sp.*	Fruits	Crown	2013-07-22	-27.0	3.8	43.5	2.2	22.8	1	0	0	0	0
K1-F005	*Drypetes sp.*	Fruits	Crown	2013-07-23	-26.7	4.0	47.3	1.7	33.0	1	0	0	0	0

K1-F033	*Drypetes sp.*	Fruits	Crown	2013-07-29	-28.4	3.2	41.7	2.4	19.9	1	0	0	0	0
K1-F053*	*Ficus bubu*	Fruits	Crown	2013-08-08	-20.1	2.6	46.8	1.7	32.6	1	1	0	0	1
K1-F057	*Ficus bubu*	Fruits	Crown	2013-08-09	-27.9	3.1	44.8	1.0	50.9	1	1	0	0	1
K2-F001	*Ficus densistipulata*	Fruits	Crown	2015-02-23	-31.8	6.9	43.9	1.1	47.9	1	0	0	0	0
K2-F091	*Ficus densistipulata*	Fruits	Crown	2015-03-02	-31.6	3.7	43.3	0.8	64.4	1	0	0	0	0
K3-F040	*Ficus densistipulata*	Fruits	Crown	2015-11-25	-30.5	2.7	43.3	1.0	48.5	1	0	0	0	0
K3-F072	*Ficus densistipulata*	Fruits	Crown	2015-12-11	-29.6	6.1	43.5	1.0	51.2	1	0	0	0	0
K1-F007	*Ficus natalensis*	Fruits	Crown	2013-07-23	-27.4	1.9	44.1	1.2	41.8	1	0	0	0	0
K1-F047	*Ficus natalensis*	Fruits	Crown	2013-07-30	-26.1	3.3	45.1	1.2	44.8	1	0	0	0	0
K1-F048	*Ficus natalensis*	Fruits	Crown	2013-07-30	-27.5	2.5	43.9	1.1	47.1	1	0	0	0	0
K1-F051	*Ficus natalensis*	Fruits	Crown	2013-08-08	-28.5	2.8	49.1	0.9	60.7	1	0	0	0	0
K2-F090	*Ficus natalensis*	Fruits	Crown	2015-03-02	-27.9	4.3	46.2	1.2	47.0	1	0	0	0	0
K2-F147	*Ficus natalensis*	Fruits	Crown	2015-03-04	-26.9	6.3	44.9	1.5	35.5	1	0	0	0	0
K3-F069	*Ficus natalensis*	Fruits	Crown	2015-12-09	-27.3	6.1	44.0	1.4	35.9	1	0	0	0	0
K3-F071	*Ficus natalensis*	Fruits	Crown	2015-12-10	-27.6	4.0	44.5	1.5	35.3	1	0	0	0	0
K2-F003	*Ficus sansibarica*	Fruits	Crown	2015-02-23	-26.9	6.6	41.2	1.3	36.0	1	1	0	1	1
K2-F119	*Ficus sansibarica*	Fruits	Crown	2015-03-04	-29.0	6.7	43.0	1.3	38.6	1	1	0	1	1
K3-F070	*Ficus sansibarica*	Fruits	Crown	2015-12-09	-30.0	4.9	43.2	1.4	36.1	1	1	0	1	1
K3-F073	*Ficus sansibarica*	Fruits	Crown	2015-12-12	-27.0	5.6	43.8	1.6	32.9	1	1	0	1	1
K1-F010	*Ficus saussureana*	Fruits	Crown	2013-07-25	-27.8	2.5	44.6	1.2	43.2	1	1	0	1	1
K1-F037	*Ficus saussureana*	Fruits	Crown	2013-07-29	-26.4	2.5	42.3	2.4	20.8	1	1	0	1	1
K1-F018	*Ficus saussureana*	Leaves	Crown	2013-07-26	-32.8	2.8	45.2	4.6	11.4	1	1	0	0	1
K1-F026	*Ficus saussureana*	Leaves	Crown	2013-07-27	-34.2	2.4	46.6	1.7	31.3	1	1	0	0	1
K1-F038	*Ficus saussureana*	Leaves	Crown	2013-07-29	-26.3	4.6	45.4	2.0	27.0	1	1	0	0	1

K1-F056	*Ficus saussureana*	Leaves	Crown	2013-08-09	-29.7	2.8	42.4	3.3	14.9	1	1	0	0	1
K1-F003	*Ficus sur*	Fruits	Crown	2013-07-22	-28.7	5.7	44.4	1.0	53.7	1	1	0	1	1
K1-F009	*Ficus sur*	Fruits	Crown	2013-07-24	-28.0	5.1	46.7	1.9	28.5	1	1	0	1	1
K1-F052	*Ficus sur*	Fruits	Crown	2013-08-08	-27.0	3.2	56.1	2.1	31.7	1	1	0	1	1
K1-F004	*Ficus thoningii*	Fruits	Crown	2013-07-22	-31.1	3.2	44.6	1.7	29.8	1	1	0	1	1
K1-F039	*Ficus thoningii*	Fruits	Crown	2013-07-29	-26.6	2.5	44.9	1.1	48.6	1	1	0	1	1
K1-F027	*Ficus vallis-choudae*	Fruits	Crown	2013-07-27	-25.4	3.7	46.2	0.7	76.0	1	0	0	0	0
K1-F028	*Ficus vallis-choudae*	Fruits	Crown	2013-07-27	-24.9	4.9	39.4	0.7	68.7	1	0	0	0	0
K2-F010	*Landolphia dawei*	Fruits	Subcanopy	2015-02-24	-27.7	2.7	49.6	1.9	31.1	1	0	0	1	0
K2-F052	*Landolphia dawei*	Fruits	Subcanopy	2015-02-27	-25.6	3.2	42.6	1.7	29.0	1	0	0	1	0
K3-F001	*Landolphia dawei*	Fruits	Subcanopy	2015-11-18	-29.7	2.3	50.5	2.9	20.4	1	0	0	1	0
K3-F005	*Landolphia dawei*	Fruits	Subcanopy	2015-11-19	-28.5	3.6	44.4	1.3	39.8	1	0	0	1	0
K2-F014	*Leptaspis zeylanica*	Seed	Understory	2015-02-26	-33.4	5.5	43.5	2.6	19.7	0	0	0	1	0
K3-F018	*Leptaspis zeylanica*	Seed	Understory	2015-11-20	-32.4	6.0	46.2	1.6	34.7	0	0	0	1	0
K3-F036	*Leptaspis zeylanica*	Seed	Understory	2015-11-24	-36.4	4.9	43.4	4.1	12.2	0	0	0	1	0
K1-F011	*Musanga leo-errerae*	Fruits	Crown	2013-07-25	-26.0	3.5	44.8	0.9	56.1	1	1	1	1	1
K1-F029	*Musanga leo-errerae*	Fruits	Crown	2013-07-27	-26.1	3.4	45.2	1.0	54.2	1	1	1	1	1
K1-F040	*Musanga leo-errerae*	Fruits	Crown	2013-07-29	-28.0	3.9	45.4	0.8	62.6	1	1	1	1	1
K1-F049	*Musanga leo-errerae*	Fruits	Crown	2013-07-30	-26.0	3.5	46.1	1.0	55.9	1	1	1	1	1
K1-F019	*Myrianthus holstii*	Leaves	Subcanopy	2013-07-26	-37.1	2.2	41.5	3.9	12.5	1	0	1	0	0
K2-F108	*Palisota manii*	Pith	Understory	2015-03-03	-33.7	3.5	37.0	4.0	10.9	0	0	0	1	0
K3-F016	*Palisota manii*	Pith	Understory	2015-11-20	-37.3	1.6	38.1	3.9	11.4	0	0	0	1	0
K1-F068	*Phytolacca dodecandra*	Fruits	Subcanopy	2013-08-14	-27.3	2.7	40.0	3.4	13.6	1	0	0	0	0
K1-F022	*Piper capensis*	Leaves	Gap	2013-07-26	-27.2	4.4	46.2	5.8	9.3	1	0	1	0	0

K2-F167	*Piper guineense*	Flower	Gap	2015-03-06	-30.1	2.1	42.8	3.5	14.2	0	0	1	1	0
K3-F031	*Piper guineense*	Flower	Gap	2015-11-21	-32.3	2.9	40.7	1.9	25.1	0	0	1	1	0
K2-F162	*Piper guineense*	Fruits	Gap	2015-03-06	-27.3	7.9	41.0	2.4	20.0	0	1	0	1	1
K3-F023	*Piper guineense*	Fruits	Gap	2015-11-20	-32.0	2.8	46.4	2.7	19.9	0	1	0	1	1
K1-F069	*Piper guineense*	Leaves	Gap	2013-08-14	-28.6	3.7	42.4	2.6	19.0	1	1	1	0	0
K1-F034	*Pseudospondias microcarpa*	Leaves	Crown	2013-07-29	-32.2	4.3	50.2	2.1	27.5	1	0	0	0	0
K1-F046	*Pseudospondias microcarpa*	Leaves	Crown	2013-07-30	-33.3	3.1	47.1	2.4	23.1	1	0	0	0	0
K1-F020	*Syzugium guineense*	Leaves	Crown	2013-07-26	-32.3	2.8	47.1	1.3	42.4	1	1	1	0	1
K1-F016	*Tetrochidium didymostemon*	Leaves	Subcanopy	2013-07-26	-31.0	7.8	43.9	5.8	8.9	1	0	0	0	0
K1-F017	*Tetrochidium didymostemon*	Leaves	Subcanopy	2013-07-26	-33.1	3.2	46.1	3.3	16.4	1	0	0	0	0
K1-F050	*Tetrochidium didymostemon*	Leaves	Subcanopy	2013-07-30	-33.3	3.4	46.2	4.3	12.4	1	0	0	0	0
K1-F012	*Trilepisium madagascariensis*	Leaves	Crown	2013-07-25	-25.8	3.5	47.2	2.2	24.7	1	0	0	1	0
K1-F044	*Trilepisium madagascariensis*	Leaves	Crown	2013-07-29	-34.5	1.6	43.7	2.8	18.1	1	0	0	1	0

Supporting Table S3. Summary of stable isotopic results of food plants from the Kalinzu Forest Reserve in different levels of vertical stratification. The carbon and nitrogen weight proportions of the samples (%C and %N) were also shown.

Part	%C		%N		$\delta^{13}C$		$\delta^{15}N$		n
	Mean	SD	Mean	SD	Mean	SD	Mean	SD	
Crown	45.0	2.0	1.8	0.8	-28.9	2.6	3.5	1.1	16
Subcanopy	43.1	3.5	3.0	1.1	-31.4	3.4	3.1	1.1	8
Understory	42.0	3.8	2.8	1.1	-34.3	1.1	3.8	1.5	3
Gap	43.9	1.9	3.7	1.5	-29.3	1.5	4.2	1.1	5

Supporting Table S4. Summary of stable isotopic results of previously reported isotope ratios of hairs from wild chimpanzees.

Sites	Rainfall	Hair	Hair				Plant					Reference
		n	$\delta^{13}C$, mean	$\delta^{13}C$, SD	$\delta^{15}N$, mean	$\delta^{15}N$, SD	n	$\delta^{13}C$, mean	$\delta^{13}C$, SD	$\delta^{15}N$, mean	$\delta^{15}N$, SD	
Chambura	950	7	-21.6	0.9	8.4	0.5	–	–	–	–	–	Loudon et al. (2016)
Kanyanchu	1500	4	-22.2	0.1	7.7	0.3	–	–	–	–	–	Loudon et al. (2016)
Kanyawara	1671	6	-21.7	0.4	6.8	0.9	–	–	–	–	–	Loudon et al. (2016)
Mainaro	1351	1	-21.8	–	7.5	–	–	–	–	–	–	Loudon et al. (2016)
Taï	1830	31	-23.6	0.6	7.3	0.6	99	-29.5	4.1	4.9	1.7	Fahy et al. (2013)
Cameroon	1700	38	-23.4	0.7	9.3	0.8	–	–	–	–	–	Macho and Lee-Thorp (2014)
Loango	2215	14	-22.8	0.5	5.0	0.4	31	-28.7	3.5	3.2	2.5	Oelze et al. (2014)
Gombe	1250	13	-21.8	0.3	3.5	0.3	–	-26.9	–	2.3	–	Schoeninger et al. (2016)
Ishasha	750	10	-21.8	0.3	6.9	2.1	2	-23.9	0.5	3.6	1.1	Schoeninger et al. (1999)
Ugalla	1012	12	-20.7	0.3	2.3	0.7	–	–	–	–	–	Schoeninger et al. (1999)
Fongoli	900	36	-20.6	0.4	2.9	0.3	65	-25.8	2.2	2.9	1.2	Sponheimer et al. (2006)
Issa	1220	11	-22.5	0.2	4.1	0.4	32	-27.6	3.3	3	2.8	van Castern et al. (2018)
Ngogo	1500	13	-23.8	0.2	7.2	0.3	34	-27.1	3.6	4.7	1.3	van Castern et al. (2018)
Kalinzu	1477	9	-23.8	0.7	6.3	0.6	24	-29.3	3.0	3.4	1.0	This study

Supporting Table S5. Stable isotopic results of hair serial sectioning of a male chimpanzee (Kobo) from the Kalinzu Forest Reserve.

ID	$\delta^{13}C$	$\delta^{15}N$	%C	%N	C/N	Length (mm)		The nearest points to the follicle		Median day before the death	Note
						From	To	Day before the death	Corresponding date		
KAA02-h1-01	-23.7	7.1	–	15.6	–	0.0	1.0	0.0	2013-07-30	-1.5	
KAA02-h1-02	-24.2	7.4	42.1	14.5	3.4	1.0	2.0	-3.0	2013-07-27	-4.5	
KAA02-h1-03	-24.5	6.8	44.6	15.2	3.4	2.0	3.0	-6.0	2013-07-24	-7.5	
KAA02-h1-04	-24.7	6.8	44.0	15.2	3.4	3.0	4.0	-9.0	2013-07-21	-10.5	
KAA02-h1-05	-24.6	6.8	45.0	15.7	3.4	4.0	5.0	-12.0	2013-07-18	-13.5	
KAA02-h1-06	-24.5	6.5	44.1	15.3	3.4	5.0	6.0	-15.0	2013-07-15	-16.5	
KAA02-h1-07	-24.3	6.7	46.3	15.9	3.4	6.0	7.0	-18.0	2013-07-12	-19.5	
KAA02-h1-08	-24.5	7.0	45.6	15.8	3.4	7.0	8.0	-21.0	2013-07-09	-22.5	
KAA02-h1-09	–	–	–	–	–	8.0	9.0	-24.0	2013-07-06	-25.5	Lost
KAA02-h1-10	–	–	–	–	–	9.0	10.0	-27.0	2013-07-03	-28.5	Lost
KAA02-h1-11	-24.6	6.6	46.3	15.6	3.5	10.0	11.5	-30.0	2013-06-30	-31.5	1.5 mm in length
KAA02-h1-12	-24.6	6.9	45.9	15.6	3.4	11.5	13.0	-34.5	2013-06-27	-34.5	1.5 mm in length
KAA02-h1-13	–	–	–	–	–	–	–	–	–	–	Not existing
KAA02-h1-14	-24.4	7.0	47.4	16.0	3.5	13.0	14.0	-39.0	2013-06-21	-40.5	
KAA02-h1-15	-24.4	7.0	47.0	15.8	3.5	14.0	15.0	-42.0	2013-06-18	-43.5	
KAA02-h1-16	-24.6	6.6	48.4	16.2	3.5	15.0	16.0	-45.0	2013-06-15	-46.5	
KAA02-h1-17	-24.6	6.7	45.5	15.5	3.4	16.0	17.0	-48.0	2013-06-12	-49.5	
KAA02-h1-18	-24.6	6.9	45.4	15.2	3.5	17.0	18.0	-51.0	2013-06-09	-52.5	
KAA02-h1-19	-24.5	7.1	42.7	14.5	3.4	18.0	19.0	-54.0	2013-06-06	-55.5	
KAA02-h1-20	-24.5	7.1	43.0	14.5	3.5	19.0	20.0	-57.0	2013-06-03	-58.5	
KAA02-h1-21	-24.5	7.2	42.9	14.5	3.4	20.0	21.0	-60.0	2013-05-31	-61.5	

KAA02-h1-22	-24.5	7.0	45.9	15.3	3.5	21.0	22.0	-63.0	2013-05-28	-64.5
KAA02-h1-23	-24.7	7.0	44.5	15.0	3.5	22.0	23.0	-66.0	2013-05-25	-67.5
KAA02-h1-24	-24.7	7.1	46.2	15.5	3.5	23.0	24.0	-69.0	2013-05-22	-70.5
KAA02-h1-25	-24.4	7.4	42.6	14.4	3.5	24.0	25.0	-72.0	2013-05-19	-73.5
KAA02-h1-26	-24.2	8.0	47.1	15.9	3.5	25.0	26.0	-75.0	2013-05-16	-76.5
KAA02-h1-27	-24.1	7.4	46.3	15.4	3.5	26.0	27.0	-78.0	2013-05-13	-79.5
KAA02-h1-28	-24.2	6.6	46.8	15.9	3.4	27.0	28.0	-81.0	2013-05-10	-82.5
KAA02-h1-29	-24.1	7.1	45.9	15.5	3.5	28.0	29.0	-84.0	2013-05-07	-85.5

Supporting Table S6. Stable isotope ratios of hair segments clumped into 5 mm segments.

Day before the death		$\delta^{13}C$	$\delta^{15}N$
From	To		
0	-15	-24.3	7.0
-15	-30	-24.4	6.7
-30	-45	-24.5	6.9
-45	-60	-24.6	6.9
-60	-75	-24.5	7.2
-75	-87	-24.2	7.3

Supporting Table S7. Stable isotopic results of individual amino acids of primate hairs from the Kalinzu Forest Reserve.

Species	ID	Ala		Gly		Val		Pro		Asp		Ser		Glu		Phe	
		Mean	SD	Mean	SD	Mean	SD	Mean	SD	Mean	SD	Mean	SD	Mean	SD	Mean	SD
Chimpanzee	KAA02	6.6	0.7	3.4	–	10.5	1.2	14.4	–	6.6	–	2.3	–	9.7	0.4	12.5	0.9
	KAA04	5.6	0.3	4.0	–	9.6	0.7	–	–	6.3	–	4.6	–	8.8	0.3	12.3	0.5
Guereza	KAA01	9.2	0.4	5.8	–	11.3	0.6	–	–	–	–	6.5	–	12.5	0.3	13.4	4.7
L’Hoest’s monkey	KAA03	11.9	0.4	9.1	–	14.7	0.2	21.5	–	11.4	–	10.3	–	15.3	0.1	13.2	0.8

Supporting Table S8. Summary of behavioral observational record of Kobo, a male chimpanzee.

Date	Day before the death			Presence	Consumption	
	From	To	Median		Plant food	Animal food
2013-04-01	-120	-121	-120.5	0	0	0
2013-04-02	-119	-120	-119.5	1	1	0
2013-04-03	-118	-119	-118.5	1	0	0
2013-04-04	-117	-118	-117.5	0	0	0
2013-04-05	-116	-117	-116.5	1	0	0
2013-04-06	-115	-116	-115.5	1	0	0
2013-04-07	-114	-115	-114.5	1	1	0
2013-04-08	-113	-114	-113.5	0	0	0
2013-04-09	-112	-113	-112.5	0	0	0
2013-04-10	-111	-112	-111.5	1	1	0
2013-04-11	-110	-111	-110.5	0	0	0
2013-04-12	-109	-110	-109.5	0	0	0
2013-04-13	-108	-109	-108.5	0	0	0
2013-04-14	-107	-108	-107.5	1	1	0
2013-04-15	-106	-107	-106.5	0	0	0
2013-04-16	-105	-106	-105.5	0	0	0
2013-04-17	-104	-105	-104.5	1	1	0
2013-04-18	-103	-104	-103.5	0	0	0
2013-04-19	-102	-103	-102.5	1	1	0
2013-04-20	-101	-102	-101.5	1	1	0
2013-04-21	-100	-101	-100.5	1	1	0
2013-04-22	-99	-100	-99.5	0	0	0
2013-04-23	-98	-99	-98.5	1	1	0
2013-04-24	-97	-98	-97.5	0	0	0
2013-04-25	-96	-97	-96.5	1	1	0
2013-04-26	-95	-96	-95.5	1	1	0
2013-04-27	-94	-95	-94.5	1	1	0
2013-04-28	-93	-94	-93.5	0	0	0
2013-04-29	-92	-93	-92.5	0	0	0
2013-04-30	-91	-92	-91.5	0	0	0
2013-05-01	-90	-91	-90.5	0	0	0
2013-05-02	-89	-90	-89.5	0	0	0

2013-05-03	-88	-89	-88.5	0	0	0
2013-05-04	-87	-88	-87.5	1	1	0
2013-05-05	-86	-87	-86.5	0	0	0
2013-05-06	-85	-86	-85.5	0	0	0
2013-05-07	-84	-85	-84.5	0	0	0
2013-05-08	-83	-84	-83.5	0	0	0
2013-05-09	-82	-83	-82.5	0	0	0
2013-05-10	-81	-82	-81.5	0	0	0
2013-05-11	-80	-81	-80.5	0	0	0
2013-05-12	-79	-80	-79.5	0	0	0
2013-05-13	-78	-79	-78.5	0	0	0
2013-05-14	-77	-78	-77.5	0	0	0
2013-05-15	-76	-77	-76.5	0	0	0
2013-05-16	-75	-76	-75.5	1	1	0
2013-05-17	-74	-75	-74.5	1	1	0
2013-05-18	-73	-74	-73.5	0	0	0
2013-05-19	-72	-73	-72.5	0	0	0
2013-05-20	-71	-72	-71.5	0	0	0
2013-05-21	-70	-71	-70.5	0	0	0
2013-05-22	-69	-70	-69.5	1	1	0
2013-05-23	-68	-69	-68.5	0	0	0
2013-05-24	-67	-68	-67.5	0	0	0
2013-05-25	-66	-67	-66.5	1	1	0
2013-05-26	-65	-66	-65.5	0	0	0
2013-05-27	-64	-65	-64.5	0	0	0
2013-05-28	-63	-64	-63.5	0	0	0
2013-05-29	-62	-63	-62.5	0	0	0
2013-05-30	-61	-62	-61.5	1	1	0
2013-05-31	-60	-61	-60.5	0	0	0
2013-06-01	-59	-60	-59.5	0	0	0
2013-06-02	-58	-59	-58.5	0	0	0
2013-06-03	-57	-58	-57.5	0	0	0
2013-06-04	-56	-57	-56.5	0	0	0
2013-06-05	-55	-56	-55.5	0	0	0
2013-06-06	-54	-55	-54.5	0	0	0

2013-06-07	-53	-54	-53.5	0	0	0
2013-06-08	-52	-53	-52.5	0	0	0
2013-06-09	-51	-52	-51.5	0	0	0
2013-06-10	-50	-51	-50.5	0	0	0
2013-06-11	-49	-50	-49.5	1	1	1
2013-06-12	-48	-49	-48.5	0	0	0
2013-06-13	-47	-48	-47.5	0	0	0
2013-06-14	-46	-47	-46.5	1	1	0
2013-06-15	-45	-46	-45.5	0	0	0
2013-06-16	-44	-45	-44.5	0	0	0
2013-06-17	-43	-44	-43.5	0	0	0
2013-06-18	-42	-43	-42.5	0	0	0
2013-06-19	-41	-42	-41.5	0	0	0
2013-06-20	-40	-41	-40.5	0	0	0
2013-06-21	-39	-40	-39.5	0	0	0
2013-06-22	-38	-39	-38.5	0	0	0
2013-06-23	-37	-38	-37.5	0	0	0
2013-06-24	-36	-37	-36.5	0	0	0
2013-06-25	-35	-36	-35.5	1	1	0
2013-06-26	-34	-35	-34.5	1	1	0
2013-06-27	-33	-34	-33.5	0	0	0
2013-06-28	-32	-33	-32.5	0	0	0
2013-06-29	-31	-32	-31.5	0	0	0
2013-06-30	-30	-31	-30.5	0	0	0
2013-07-01	-29	-30	-29.5	0	0	0
2013-07-02	-28	-29	-28.5	0	0	0
2013-07-03	-27	-28	-27.5	0	0	0
2013-07-04	-26	-27	-26.5	0	0	0
2013-07-05	-25	-26	-25.5	0	0	0
2013-07-06	-24	-25	-24.5	0	0	0
2013-07-07	-23	-24	-23.5	0	0	0
2013-07-08	-22	-23	-22.5	0	0	0
2013-07-09	-21	-22	-21.5	0	0	0
2013-07-10	-20	-21	-20.5	0	0	0
2013-07-11	-19	-20	-19.5	1	0	0

2013-07-12	-18	-19	-18.5	1	1	0
2013-07-13	-17	-18	-17.5	1	1	0
2013-07-14	-16	-17	-16.5	0	0	0
2013-07-15	-15	-16	-15.5	1	1	0
2013-07-16	-14	-15	-14.5	1	0	0
2013-07-17	-13	-14	-13.5	1	0	0
2013-07-18	-12	-13	-12.5	1	1	0
2013-07-19	-11	-12	-11.5	1	1	0
2013-07-20	-10	-11	-10.5	1	1	0
2013-07-21	-9	-10	-9.5	0	0	0
2013-07-22	-8	-9	-8.5	1	1	0
2013-07-23	-7	-8	-7.5	1	1	0
2013-07-24	-6	-7	-6.5	1	1	0
2013-07-25	-5	-6	-5.5	1	1	0
2013-07-26	-4	-5	-4.5	1	0	0
2013-07-27	-3	-4	-3.5	0	0	0
2013-07-28	-2	-3	-2.5	0	0	0
2013-07-29	-1	-2	-1.5	0	0	0
2013-07-30	0	-1	-0.5	0	0	0

Supporting References

Fahy, G. E., Richards, M., Riedel, J., Hublin, J.-J., & Boesch, C. (2013). Stable isotope evidence of meat eating and hunting specialization in adult male chimpanzees. Proceedings of the National Academy of Sciences, 110, 5829–5833.

Loudon, J. E., Sandberg, P. A., Wrangham, R. W., Fahey, B., & Sponheimer, M. (2016). The stable isotope ecology of *Pan* in Uganda and beyond. American Journal of Primatology, 78, 1070–1085.

Macho, G. A, & Lee-Thorp, J. A. (2014). Niche partitioning in sympatric gorilla and pan from Cameroon: implications for life history strategies and for reconstructing the evolution of hominin life history. PloS One, 9, e102794.

Oelze, V. M., Head, J. S., Robbins, M. M., Richards, M., & Boesch, C. (2014). Niche differentiation and dietary seasonality among sympatric gorillas and chimpanzees in Loango National Park (Gabon) revealed by stable isotope analysis. Journal of Human Evolution, 66, 95–106.

Schoeninger, M. J., Moore, J., & Sept, J. M. (1999). Subsistence strategies of two “savanna” chimpanzee populations: the stable isotope evidence. American Journal of Primatology, 49, 297–314.

Schoeninger, M. J., Most, C. A., Moore, J. J., & Somerville, A. D. (2016). Environmental variables across *Pan troglodytes* study sites correspond with the carbon, but not the nitrogen, stable isotope ratios of chimpanzee hair. American Journal of Primatology, 78, 1055–1069.

Sponheimer, M., Loudon, J. E., Codron, D., Howells, M. E., Pruetz, J. D., Codron, J., … Lee-Thorp, J. A. (2006). Do “savanna” chimpanzees consume C_4 resources? Journal of Human Evolution, 51, 128–133.

van Casteren, A., Oelze, V. M., Angedakin, S., Kalan, A. K., Kambi, M., Boesch, C., … Kupczik, K. (2018). Food mechanical properties and isotopic signatures in forest versus savannah dwelling eastern chimpanzees. Communications Biology, 1, 109.
